# Validation of three predictive models for suboptimal cytoreductive surgery in advanced ovarian cancer

**DOI:** 10.1038/s41598-021-86928-2

**Published:** 2021-04-14

**Authors:** Antoni Llueca, María Teresa Climent, Javier Escrig, Paula Carrasco, Anna Serra, MUAPOS working group (Multidisciplinary Unit of Abdominal Pelvic Oncology Surgery), L. Gomez-Quiles, R. Játiva, G. Cebrian, V. Bosso, A. Villarin, K. Maiocchi, K. Delgado-Barriga, M. Rodrigo-Aliaga, N. Ruiz, C. Herrero, A. Frances, I. Beato, C. Ferrer, J. P. Aracil, E. Boldo, A. Boldo, R. Adell

**Affiliations:** 1 Multidisciplinary Unit of Abdominal Pelvic Oncology Surgery (MUAPOS), University General Hospital of Castellon, Av Benicasim s/n, 12004 Castellón, Spain; 2 Department of Medicine, University Jaume I (UJI), Castellón, Spain; 3 Department of Oncology, Consorci Hospitalari Provincial de Castello, Castellón, Spain; 4 Department of Radiotherapy, Consorci Hospitalari Provincial de Castello, Castellón, Spain; 5 Department of Plastic Surgery, Consorci Hospitalari Provincial de Castello, Castellón, Spain; 6 Department of Surgery, Consorci Hospitalari Provincial de Castello, Castellón, Spain; 7 Department of Obsterics and Gynecology, La Plana University Hospital, Castellón, Spain; 8 Department of Surgery, La Plana University Hospital, Castellón, Spain

**Keywords:** Oncology, Surgical oncology

## Abstract

The standard treatment for advanced ovarian cancer (AOC) is cytoreduction surgery and adjuvant chemotherapy. Tumor volume after surgery is a major prognostic factor for these patients. The ability to perform complete cytoreduction depends on the extent of disease and the skills of the surgical team. Several predictive models have been proposed to evaluate the possibility of performing complete cytoreductive surgery (CCS). External validation of the prognostic value of three predictive models (Fagotti index and the R3 and R4 models) for predicting suboptimal cytoreductive surgery (SCS) in AOC was performed in this study. The scores of the 3 models were evaluated in one hundred and three consecutive patients diagnosed with AOC treated in a tertiary hospital were evaluated. Clinicopathological features were collected prospectively and analyzed retrospectively. The performance of the three models was evaluated, and calibration and discrimination were analyzed. The calibration of the Fagotti, R3 and R4 models showed odds ratios of obtaining SCSs of 1.5, 2.4 and 2.4, respectively, indicating good calibration. The discrimination of the Fagotti, R3 and R4 models showed an area under the ROC curve of 83%, 70% and 81%, respectively. The negative predictive values of the three models were higher than the positive predictive values for SCS. The three models were able to predict suboptimal cytoreductive surgery for advanced ovarian cancer, but they were more reliable for predicting CCS. The R4 model discriminated better because it includes the laparotomic evaluation of the peritoneal carcinomatosis index.

## Introduction

Ovarian cancer is one of the highest mortality cancers among gynecologic cancers worldwide. They are frequently diagnosed in advanced stages, after the disease has spread throughout the abdominal cavity. Standard treatment of epithelial ovarian cancer includes staging and/or cytoreduction of the disease along with adjuvant platinum-based chemotherapy^1^.

Some studies have indicated that one of the major prognostic factors that affect the survival of patients with advanced ovarian cancer (AOC) is the residual tumor after primary cytoreduction surgery (PDS)—specifically, for microscopic or < 1 cm residual tumor, survival was higher than for > 1 cm residual tumor, called optimal cytoreduction surgery (OCS) and suboptimal cytoreduction surgery (SCS), respectively. Currently, the primary goal of PDS has changed to leave at most a microscopic residual tumor, also called complete cytoreductive surgery (CCS)^2–5^.

However, not all patients can undergo such aggressive surgery to achieve optimal cytoreduction at the time of diagnosis. Some authors have shown that neoadjuvant chemotherapy (NAC) in these cases does not imply a worse prognosis in these patients and significantly reduces postoperative complications that may become high^6^. Other authors have discussed these studies, arguing that although the optimal coreduction rates in adjuvant cases were higher than those in PDS, they were far from those described in other published studies. Even so, patients with optimal cytoreduction in PDS had a better prognosis in the majority of studies^7^. Nevertheless, NAC has been proposed as an alternative for patients with AOC with an excessively high fragility index for PDS^8,9^.

For this reason, clinical predictive models are needed to aid in the personalized selection of patients with AOC. To more accurately quantify the intra-abdominal extension of AOC, multiple predictive models and classification systems have been proposed^10–15^, among which clinical models that indicate the risk of performing suboptimal cytoreduction surgery are worth noting due to their involvement in the management of these patients. One of the best known and used models is the Fagotti model, as well as ours, the R3 and R4 models^14,15^.

The objective of the study was to analyze and validate the prognostic performance of these three models (Fagotti, R3, and R4) in predicting SCS in a sample of patients with advanced ovarian cancer.

## Methods

One hundred and three consecutive patients with AOC were treated at the Multidisciplinary Unit of Abdominal Pelvic Oncology Surgery (MUAPOS) at the University General Hospital of Castellon, Spain, from January 2015 to March 2020. Age and health status were also considered. The same surgical team performed all procedures, including the collection of the data describing patients' clinical and pathological characteristics, surgical procedures, and residual disease at surgery. These data were collected prospectively and analyzed retrospectively. Informed consent was obtained from all patients, and the study was approved by the Committee on Ethics and Research on Drugs of the University General Hospital of Castellon, Spain, who confirmed that all investigations were conducted in accordance with relevant guidelines/regulations.

The scores of the three models (Table 1) were obtained from a consecutive sample of patients with recurrent and primary AOC, leading to a final score (total score) for each patient targeted to predict SCS. The Fagotti model obtains all its scores from diagnostic laparoscopy; the R3 model score is obtained from the findings of preoperative thoracoabdominal computed tomography (CT), from the calculation of laparoscopic PCI, and the presence of clinical or radiology of partial bowel obstruction. The R4 model adds the operative PCI to the result of the R3 model^15^.

**Table 1:** Model scores.

FAGOTTI model scores	Presence	Absence
Omental cake	2	0
Peritoneal carcinomatosis	2	0
Diaphragmatic carcinomatosis	2	0
Mesenteric retraction	2	0
Small bowel/Stomach implants	2	0
Glisson implants	2	0
**R3 model scores**		
CT scan PCI ≥ 20	1	0
Laparoscopic PCI ≥ 20	1	0
Partial Bowel obstruction	2	0
**R4 model scores**		
CT scan PCI ≥ 20	1	0
Laparoscopic PCI ≥ 20	1	0
Partial Bowel obstruction	2	0
Operatory PCI ≥ 20	2	0

All patients underwent surgery intending to achieve complete or optimal cytoreduction, which was assessed at the end of the intervention according to the following criteria. Two experienced gynecological oncologists recorded intraoperative assessments of tumor burden with a peritoneal cancer index (PCI) score^12^. Residual disease after surgery was reported as complete cytoreductive surgery (CCS), optimal cytoreductive surgery (OCS) and suboptimal cytoreductive surgery (SCS). CCS was defined as the absence of macroscopic residual disease. OCS and SCS were defined as macroscopic residual disease with a maximal diameter < 1 cm and 1 > cm, respectively. For the aim of this study, optimal resection was defined as as the combination of CCS and OCS. For statistical purposes, the results of cytoreductive surgery were divided into two groups: OCS (complete/optimal cytoreductive surgery) and SCS (suboptimal cytoreductive surgery).

For each of the models, their discrimination and calibration capabilities were analyzed by comparing this binary outcome variable with the final score of each case. The achievement of SCS was considered as an event.

To analyze the calibration of the models, the odds ratios of each model and their respective total scores were obtained by means of binary logistic regression as a measure of the increase in risk and derived from a point increase in the total score, as well as by the Hosmer–Lemeshow test to compare differences between observed events and events predicted (expected) by the model. With the probabilities of observed events and expected events, calibration graphs were made by grouping the probabilities. The diagnostic accuracy of each model was calculated by calculating the percentage of correct SCS predictions.

To analyze the discrimination of the models, the area under the ROC curve (AUC) was calculated, and the optimal cutoff value of the total scores of each model was obtained, which determined the best combination of sensitivity and specificity. Finally, the AUCs of the model were compared. Statistical analysis was performed using Stata Statistical Software Release 16 (College Station, TX: StataCorp LLC). The calibration graphs were made with the "user-writer" *pmcalplot* command.

## Results

A total of 103 patients with confirmed AOC were included in this study. All of them were eligible for primary or secondary cytoreductive surgery. None of the patients included in this study met our Radiologic-Laparoscopic Criteria for Unresectability (RLCU)^13^.

The patient characteristics are summarized in Table 2, and the surgical procedures are shown in the supplementary materials. The median age (range) was 60 (51—67) years. The median levels of CA 12.5 and CA 19.9 were 924.3 and 372.7 IU/mL, respectively. Seventy-nine patients (76.7%) were in FIGO stage III, and 71% of the patients had high-grade serous ovarian cancer.

**Table 2:** Characteristics of the patients.

	Total N = 103	OCS N = 87	SCS N = 16	*p* value
**Age**	60 (51–67)	60 (51–67)	63 (55–67)	0.51
**Current tumor**				
Primary	74 (71.8%)	63 (72.4%)	11 (68.8%)	0.76
Recurrent	29 (28.2%)	24 (27.6%)	5 (31.2%)	
**FIGO stage**				
III	79 (76.7%)	72 (82.8%)	7 (43.8%)	< 0.001
IV	24 (23.3%)	15 (17.2%)	9 (56.2%)	
**Histology**				
Serous	71 (71%)	62 (72.9%)	9 (60%)	0.097
Adenocarcinoma	8 (8%)	4 (4.7%)	4 (26.7%)	
Mucinous	2 (2%)	2 (2.4%)	0	
Clear cells	3 (3%)	2 (2.4%)	1 (6.7%)	
Endometroid	7 (7%)	6 (7.1%)	1 (6.7%)	
Undifferentiated	5 (5%)	5 (5.9%)	0	
Other	4 (4%)	4 (4.7%)	0	
**Charlson age index**				
	2 (0–3)	2 (0–3)	2 (1–4)	0.40
**Ascites in imaging tests**				
No	66 (64.1%)	59 (67.8%)	7 (43.8%)	0.06
Yes	37 (35.9%)	28 (32.2%)	9 (56.2%)	
**Intestinal occlusion in imaging tests**				
No	97 (94.2%)	83 (95.4%)	14 (87.5%)	0.21
Yes	6 (5.8%)	4 (4.6%)	2 (12.5%)	
**Preoperative malnutrition**				
No	78 (75.7%)	67 (70%)	11 (68.8%)	0.48
Yes	25 (24.3%)	20 (23%)	5 (31.2%)	
**Total CT scan PCI**				
	12 (5–18)	11 (4–15)	17 (11–25)	0.004
**Categorized CT scan PCI**				
1–10	50 (51%)	46 (55%)	4 (25%)	0.02
11–20	36 (36%)	29 (35%)	7 (44%)	
20	13 (13%)	8 (10%)	5 (31%)	
**Total operative PCI**				
	14 (7–20)	12 (5–15)	26 (21–33)	< 0.001
**Categorized operative PCI**				
1–10	41 (40.6%)	40 (47.1%)	1 (6.2%)	< 0.001
11–20	35 (34.7%)	32 (37.6%)	3 (18.8%)	
20	25 (24.8%)	13 (15.3%)	12 (75%)	

*Data are presented as n (%) and median (interquartile range).

OCS and SCS were achieved in 87 (84.5%) and 16 (15.5%) patients, respectively. The mean surgical time was 420 min, including the anesthesia induction time. Visceral resections were performed in 85 (82.5%) patients. Major complications (grade III and IV from the Clavien-Dindo classification) were found in 29 (28%) patients.

### Performance of the predictive models for SCS

A descriptive analysis of the total scores obtained with each model is shown in Table 3.

**Table 3:** Descriptive analysis of the total scores obtained with each model.

	Scores maximum range observed	Total n = 103 scores mean ± SD	SCS n = 16 sores mean ± SD	OCS n = 87 scores mean ± SD
FAGOTTI	0–10	4 ± 3	7 ± 2	3 ± 3
R3	0–3	0.6 ± 1	2 ± 2	0.5 ± 1
R4	0–4	1 ± 2	3 ± 2	1 ± 1

SD, standard deviation.

The calibration analysis is shown in Table 4. The odds ratio indicates the increased risk of SCS for each additional point in the model total score. The Hosmer–Lemeshow calibration test analyzes the number of predicted cases of SCS with respect to the observed cases. The test yielded no significant p-values, which indicates good calibration in the three models. The R4 model has the highest percentage of SCS cases predicted.

**Table 4:** Calibration analysis. Logistic regression: SCS and OCS versus Models scores.

	Odds ratio*	95% CI odds ratio	Odds ratio *p* value	Hosmer–Lemeshow *p* value	Correct classification (%)
FAGOTTI	1.6	1.2–2.1	< 0.001	0.995	86
R3	2.4	1.4–4	< 0.001	0.705	87
R4	2.4	1.5–3.7	< 0.001	0.852	91

*Increased risk of suboptimal cytoreduction for each additional point.

**Figure 1:**
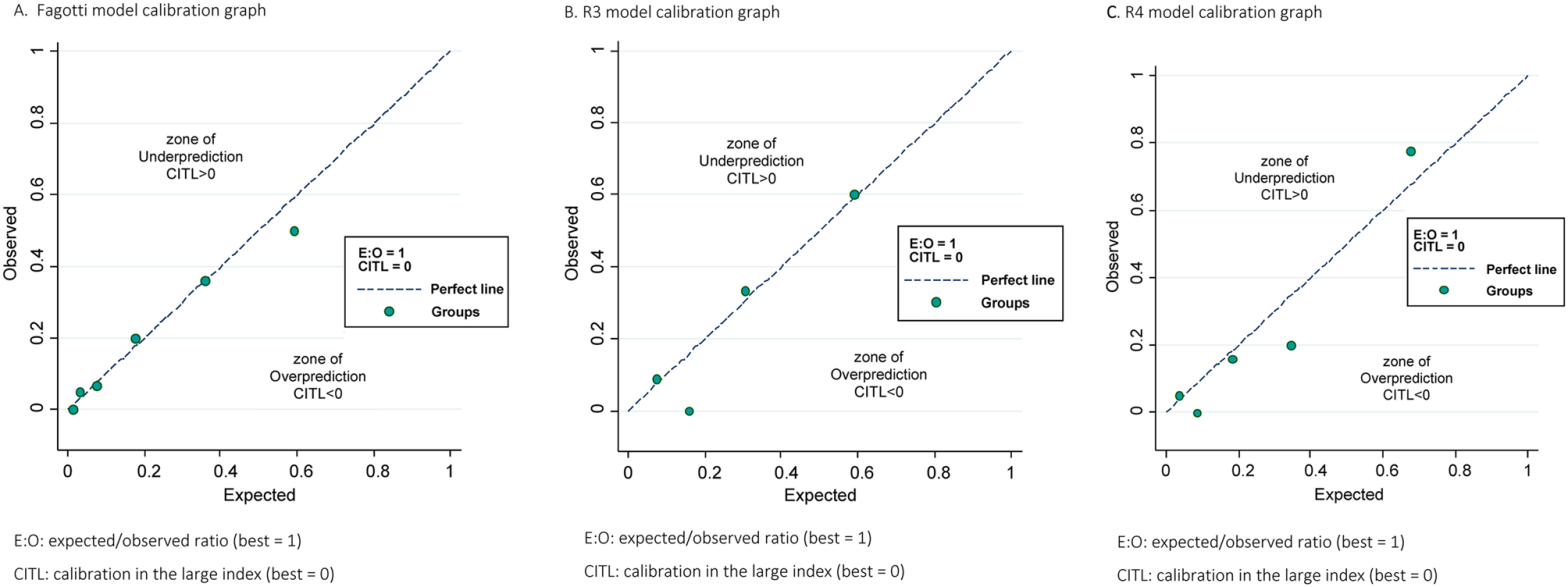
Calibration plots for suboptimal cytoreduction by probability groups (**A**) Fagotti model calibration graph. E:O: expected/observed ratio (best = 1), CITL: calibration in the large index (best = 0). (**B**) R3 model calibration graph. E:O: expected/observed ratio (best = 1). CITL: calibration in the large index (best = 0). (**C**) R4 model calibration graph. E:O: expected/observed ratio (best = 1). CITL: calibration in the large index (best = 0).

Figure 1 shows the three calibration plots by groups of probabilities for expected and observed cases of SCS. These figures graphically represent the calibration of the models in terms of probability ranges. All circles referring to the probability groups fall near the diagonal line, indicating a correct calibration for the three models. In the same sense, the expected/observed ratio has a value of 1, and the calibration in the large (CITL) index has a value of 0 for all models.

The discrimination analysis is shown in Table 5.

**Table 5:** Discrimination analysis. Areas Under the ROC Curves.

	AUC^1^	95% CI AUC^2^	Optimal cutoff	Sensitivity (%)	Specificity (%)
FAGOTTI	0.83	0.74–0.90	< 4	86	74
R3	0.70	0.60–0.79	< 1	57	86
R4	0.81	0.72–0.88	< 1	79	74

^1^Hanley-McNeil.

^2^Exact binomial.

Prevalence suboptimal cytoreduction: 15%.

Fagotti: SCS Positive predictive value: 37%; SCS Negative predictive value: 97%.

R3: SCS Positive predictive value: 42%; SCS Negative predictive value: 92%.

R4: SCS Positive predictive value: 35%; SCS Negative predictive value: 95%.

**Figure 2:**
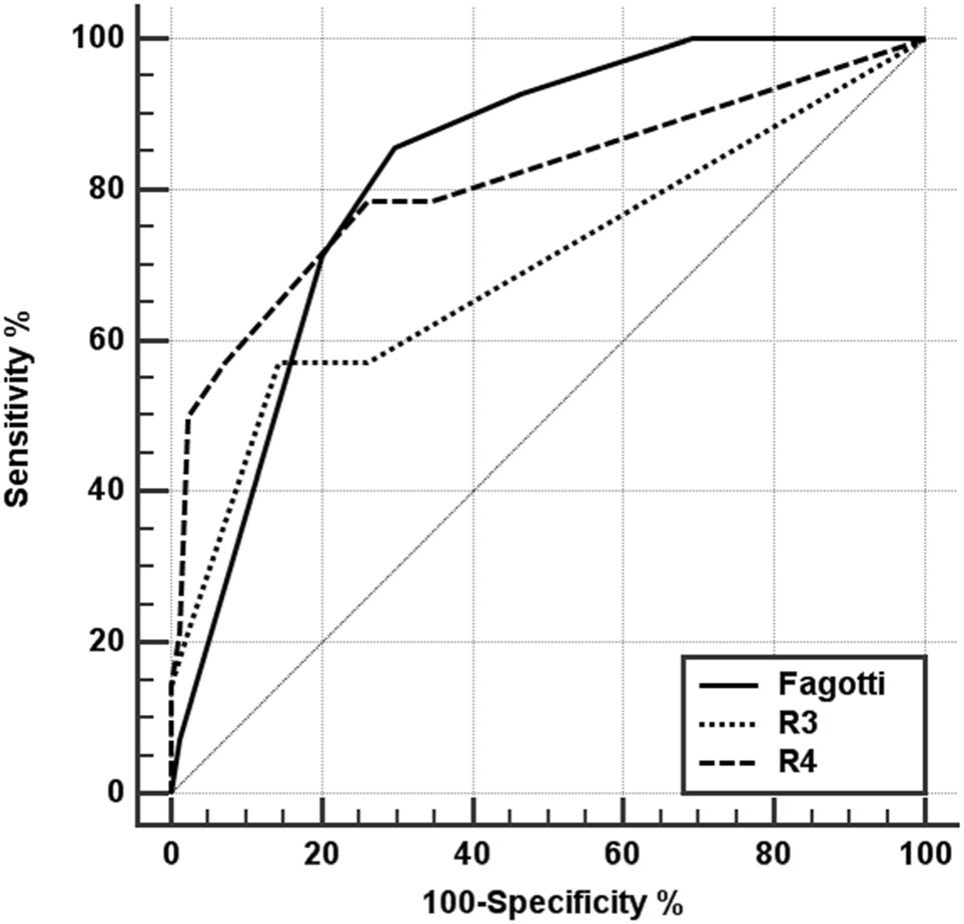
Comparative graph of the ROC curves for the different models.

These areas are graphically compared in Fig. 2, which shows good discrimination and few differences between the three models analyzed. According to the optimal cutoff model score as determined by the ROC curves, the values found for sensitivity and specificity indicate that in contexts of low SCS prevalence (5–25%), the three models offer negative predictive values far superior to the positive predictive values for SCS (Table 6). Therefore, these models are more reliable in predicting optimal surgery than in predicting suboptimal surgery when the total score is equal to or less than the optimal cutoff.

**Table 6:** Differences in Areas Under ROC Curves.

AUC difference (*p* value)*	FAGOTTI	R3	R4
FAGOTTI	–	0.13 (0.173)	0.02 (0.849)
R3	− 0.13 (0.173)	–	− 0.11 (0.013)
R4	− 0.02 (0.849)	0.11 (0.013)	–

*Reading: row versus column.

Table 6 shows that the differences in terms of discrimination between the 3 models are low and not significant except between the R3 and R4 models.

## Discussion

The present study validated three predictive models for SCS in a prospective and consecutive cohort of patients treated by a surgical team specializing in advanced ovarian cancer surgery. The application of the predictive models in the clinic would allow better classification of patients in different treatment approaches, achieving high OCS rates with acceptable morbidity.

The tumor volume after surgery for advanced ovarian cancer is inversely related to survival, defining the gold standard of ovarian cancer surgery as complete cytoreduction and the absence of tumors^2,16^.

From the defining of these concepts were defined until now, surgical teams have been improving their surgical techniques, achieving high cytoreduction rates^17,18^.

Surgical techniques of the upper abdomen have been used to achieve cytoreduction percentages greater than 47%, and their use is related to an increase in the cytoreduction rate but possibly also to an increase in morbidity^19,20^. Establishing a balance between optimal cytoreduction and the number of complications constitutes the key point in the current situation for surgical teams specializing in advanced ovarian cancer surgery.

The ability to perform optimal cytoreduction is first determined by the extent of the disease, for which several predictive models have been proposed^12–15^.

Preoperative radiological evaluation of these patients constitutes the most frequently used primary resource^13,21–24^. Some authors have reported good results with only the use of radiologic imaging, but these results have not been validated in other institutions^25,26^. Consequently, the preoperative radiological signs of disease extension should be used with caution, especially when they are used exclusively in making decisions about the outcome of cytoreduction surgery.

Laparoscopic minimally invasive techniques have been used for many years to facilitate biopsies and assess COC resectability. There are several methods that are used in the clinic for the evaluation of the disease; among them, the most commonly used are those of Fagotti and the PCI^12,14^. The Fagotti model was designed by his surgical team when they reported an OCS rate of 67% with a concordance between laparoscopy and the final result of surgery of 69% and a rate of unnecessary laparotomies of 34%. Later, Petrillo updated the Fagotti score by introducing upper abdominal surgical techniques, reporting OCS percentages of approximately 80%, but even so, they did not manage to reduce the percentage of unnecessary laparotomies, which remained at 33%. This model was also externally validated by Cherau and Feng, who reported area under the curve (AUC) values of 51% and 71%, respectively, in their series when discriminating between OCS and SCS^25–28^.

The difficulty of laparoscopy in AOC lies in its difficulty in evaluating the deeper regions of the abdomen, which may be the reason for surgical unresectability. The regions of the liver pedicle, the retrohepatic region, or the retroperitoneal space can be difficult if not impossible to assess using the laparoscopic approach. For this reason, we must use imaging techniques that evaluate these regions with more precision and can help us avoid unnecessary laparotomies and reduce the percentage of SCS^29,30^. Therefore, we described two models that combine both laparoscopic and radiological images (model R3) and, later, laparotomic findings (model R4)^15^.

When we externally validated these models on a consecutive and different sample of patients and compared it with a widely used model such as that of Fagotti, we affirmed that the three models work correctly, without major differences when predicting SCS for advanced ovarian cancer. Perhaps the R4 model discriminates better because it includes a laparotomic evaluation of the PCI index at the time of surgery.

Nevertheless, there are some differences between the R3-R4 models and the Fagotti model. First, we used the PCI, which provided information to the R3 and R4 models in the form of the quantification of tumor volume by region that helped us more objectively categorize the advanced stages of FIGO for ovarian cancer. Furthermore, the value of the categorized PCI revealed PCI category as an independent prognostic factor, with significantly better survival rates in patients with PCI ⩽10 compared with either PCI 11–20 or > 20, with 5-year survival rates of 70%, 30%, and 28%, respectively.

From a mathematical point of view, in the validation sample, the three models analyzed achieved correct discrimination and calibration for SCS prediction. In practice, the final diagnostic performance of a clinical model is determined by predictive values, which are acceptable when equal to or greater than 80%. The sensitivity and specificity of a diagnostic test reflect the diagnostic capacity inherent within it. However, the final diagnostic performance corresponds to the predictive values, which are determined by the prevalence of the disease based on its sensitivity and specificity. In this sense, the three models demonstrated greater reliability in predicting optimal surgery (higher negative predictive values for SCS) in situations in which a high optimal cytoreduction rate is achieved by experienced surgical teams, that is, where the prevalence of SCS is low. It may seem paradoxical that models designed to predict suboptimal surgery work best when their scores are low in magnitude. This is a consequence of the conditional probability laws that govern the results of diagnostic tests.

One of the strengths of this work is that it was carried out in a cohort of consecutive patients managed according to the same protocols for a limited period of time and supervised by the same surgical team in a university hospital setting with a unit dedicated to the treatment of these patients. In addition, all the models were adjusted for the optimal and suboptimal debulking rate of our unit, which independently describes the behavior of the different models in a controlled environment under the same conditions. Another strength, and one of the differences compared to other external evaluations, is the fact that in this work, an attempt is made to achieve CCS, not OCS, because in surgical teams with high specialization and high optimal cytoreduction rates of above 70%, it is indeed difficult to decide whether to perform a laparotomy, since generally, they will achieve satisfactory results. The limitations of the study are its retrospective nature and the limited number of patients. Prospective external validation of the R3 and R4 models in the PREdictive Models in Ovarian Cancer Surgery (PREMOCS) trial is ongoing at this moment, and we expect to see results in the near future.

In conclusion, the three models analyzed are useful in the prediction of SCS from a statistical perspective, with few differences between them but demonstrating high reliability when predicting complete surgery or optimal surgery.

## Supplementary Information

Supplementary Information

## Data Availability

The data that support the findings of this study are available from the corresponding author upon reasonable request.

## Abbreviations

AOC: Advanced ovarian cancer
CCS: Complete cytoreductive surgery
CT: Computed tomography
MUAPOS: Multidisciplinary unit of abdominal pelvic oncology surgery
NAC: Neoadjuvant chemotherapy
OCS: Optimal cytoreduction surgery
PCI: Peritoneal cancer index
PDS: Primary cytoreduction surgery
ROC curve: Receiver operating characteristic curve
RLCU: Radiologic-laparoscopic criteria for unresectability
SCS: Suboptimal cytoreductive surgery
