# Supplementary material for: Validation of three predictive models for suboptimal cytoreductive surgery in advanced ovarian cancer
Source: Sci Rep. 2021 Apr 14;11:8111. doi: 10.1038/s41598-021-86928-2 (PMC8047030; doi:10.1038/s41598-021-86928-2)
Supplement: Supplementary file 1 — Supplementary Information [file 41598_2021_86928_MOESM1_ESM.docx]

**Table 3.** Surgical procedures.

|  |  | **Total** | **OCS** | **SCS** | **p-value** |
| --- | --- | --- | --- | --- | --- |
|  |  | **N=103** | **N=87** | **N=16** |  |
| Duration of surgery (min) |  | 420 (300-570) | 420 (300-580) | 308 (230-555) | 0.15 |
| Ascites | No | 74 (71.8%) | 69 (79.3%) | 5 (31.2%) | <0.001 |
|  | Yes | 29 (28.2%) | 18 (20.7%) | 11 (68.8%) |  |
| Visceral resections | No | 18 (17.5%) | 12 (13.8%) | 6 (37.5%) | 0.022 |
|  | Yes | 85 (82.5%) | 75 (86.2%) | 10 (62.5%) |  |
| Pelvic peritonectomy | No | 37 (35.9%) | 30 (34.5%) | 7 (43.8%) | 0.48 |
|  | Yes | 66 (64.1%) | 57 (65.5%) | 9 (56.2%) |  |
| Left diaphragm  peritonectomy | No | 68 (66%) | 57 (65.5%) | 11 (68.8%) | 0.80 |
|  | Yes | 35 (34%) | 30 (34.5%) | 5 (31.2%) |  |
| Right diaphragm peritonectomy | No | 58 (56.3%) | 48 (55.2%) | 10 (62.5%) | 0.59 |
|  | Yes | 45 (43.7%) | 39 (44.8%) | 6 (37.5%) |  |
| Mes jenunal peritonectomy | No | 92 (89.3%) | 77 (88.5%) | 15 (93.8%) | 0.53 |
|  | Yes | 11 (10.7%) | 10 (11.5%) | 1 (6.2%) |  |
| Hilium hepatic peritonectomy | No | 74 (71.8%) | 63 (72.4%) | 11 (68.8%) | 0.76 |
|  | Yes | 29 (28.2%) | 24 (27.6%) | 5 (31.2%) |  |
| Rectal resection | No | 71 (68.9%) | 62 (71.3%) | 9 (56.2%) | 0.23 |
|  | Yes | 32 (31.1%) | 25 (28.7%) | 7 (43.8%) |  |
| Sigma resection | No | 63 (61.2%) | 55 (63.2%) | 8 (50%) | 0.32 |
|  | Yes | 40 (38.8%) | 32 (36.8%) | 8 (50%) |  |
| Transverse colon resection | No | 85 (82.5%) | 72 (82.8%) | 13 (81.2%) | 0.88 |
|  | Yes | 18 (17.5%) | 15 (17.2%) | 3 (18.8%) |  |
| Cecum or appendix resection | No | 63 (61.2%) | 52 (59.8%) | 11 (68.8%) | 0.50 |
|  | Yes | 40 (38.8%) | 35 (40.2%) | 5 (31.2%) |  |
| Ileum segment resection | No | 81 (78.6%) | 65 (74.7%) | 16 (100%) | 0.023 |
|  | Yes | 22 (21.4%) | 22 (25.3%) | 0 |  |
| Pancreas corporocaudal resection | No | 92 (89.3%) | 78 (89.7%) | 14 (87.5%) | 0.80 |
|  | Yes | 11 (10.7%) | 9 (10.3%) | 2 (12.5%) |  |
| Spleen resection | No | 76 (73.8%) | 65 (74.7%) | 11 (68.8%) | 0.62 |
|  | Yes | 27 (26.2%) | 22 (25.3%) | 5 (31.2%) |  |
| Atypical hepatectomies | No | 97 (94.2%) | 84 (96.6%) | 13 (81.2%) | 0.016 |
|  | Yes | 6 (5.8%) | 3 (3.4%) | 3 (18.8%) |  |
| Glissectomy | No | 88 (85.4%) | 75 (86.2%) | 13 (81.2%) | 0.61 |
|  | Yes | 15 (14.6%) | 12 (13.8%) | 3 (18.8%) |  |
| Stoma performance | No | 87 (84.5%) | 76 (87.4%) | 11 (68.8%) | 0.059 |
|  | Yes | 16 (15.5%) | 11 (12.6%) | 5 (31.2%) |  |
| Length of Stay |  | 15 (9-30) | 15 (10-30) | 14 (8-28) | 0.88 |
| Complication Grade categorized | No | 52 (50.5%) | 47 (54%) | 5 (31.2%) | 0.24 |
|  | Minor | 22 (21.4%) | 17 (19.5%) | 5 (31.2%) |  |
|  | Major | 29 (28.2%) | 23 (26.4%) | 6 (37.5%) |  |
| Mortality 30 days | No | 99 (96.1%) | 86 (98.9%) | 13 (81.2%) | <0.001 |
|  | Yes | 4 (3.9%) | 1 (1.1%) | 3 (18.8%) |  |

Data are presented as n (%) and median (interquartile range.
